# Frequency and assessment of Nutritional Status of school going children in rural areas of Islamabad

**DOI:** 10.12669/pjms.37.5.3773

**Published:** 2021

**Authors:** Shehla Farhin, Tamkeen Jaffry, Sadia Zafar, Farah Rashid

**Affiliations:** 1 Dr. Shehla Farhin, MPH. Assistant Professor, Department of Community Medicine, Islamabad Medical and Dental College, Islamabad, Pakistan; 2 Dr. Tamkeen Jaffry, MPH. Assistant Professor, Department of Community Medicine, Islamabad Medical and Dental College, Islamabad, Pakistan; 3 Dr. Sadia Zafar, MSPH. Lecturer. Department of Community Medicine, Islamabad Medical and Dental College, Islamabad, Pakistan; 4 Dr. Farah Rashid, MPH Professor/ Head of Department, Department of Community Medicine, Islamabad Medical and Dental College, Islamabad, Pakistan

**Keywords:** Nutritional status, School children, Malnutrition, Rural Islamabad, BMI

## Abstract

**Background and Objectives:**

Nutritional status is considered a significant and positive health indicator. It determines anthropometric measurements of preschool children, the height of children at the time of school entry and prevalence of low birth weight. The objective of the study was to determine the frequency of nutritional status and socio-demographic factors influencing under nutrition among school children of rural Islamabad.

**Methods:**

A cross-sectional study was conducted among school children of the age (4-16 years) from January 2017 to September 2019. The Sample size was 1710. Schools were selected through convenient sampling technique. Frequency and percentages were calculated and inferential statistics were computed to analyze the association of health status with categorical variables by using chi-square by keeping the level of significance <0.05 through SPSS version 20.

**Results:**

The mean age of the sample was 9.38 ± 4.14 with the maximum number of children (49.1%) in the age bracket of 5-9. Out of 1710 children, 54.4% had normal weight for age, 25.3% were underweight, 7.5% overweight and 12.8% were found to be obese. Stunting was found to be 26%. Prevalence of being underweight was higher than overweight /obesity particularly in younger and higher age groups as indicated by p-value of 0.000. Comparing with females, male students had significantly higher frequency of being underweight and stunted as reflected by p-value of 0.004 and 0.000 respectively. Univariate analysis also showed a strong association between age and nutritional status as mean weight increased from 39.22 ±5.21 to 63.50± 4.66 and height from 35.67±5.76 to 113.73± 29.22 with advancing age.

**Conclusions:**

Undernutrition remains an ongoing health problem in school going children of rural Islamabad; particularly in male students of younger age groups. School health programs and nutritional interventions need to be strengthened particularly in rural areas of Islamabad.

## INTRODUCTION

Malnutrition is the biggest threat for the global burden of disease.^1^ Nutritional status is a main key for determining quality of life mainly in children. Malnutrition is a serious condition that occurs when a person’s diet does not contain enough nutrients to meet the demands of their body.

WHO estimated that 27% of the children in developing countries less than five years of age are underweight. According to National Nutrition Survey 2011, 31% children under the age of five years are underweight. The nutrition of primary school children is vital and active part for their physical and mental development.^2^,^3^

According to the World Health Organization Malnutrition is defined as “The cellular imbalance between the supply of nutrients and energy and the body’s demand for them to ensure growth, maintenance, and specific functions.” Both undernutrition and over nutrition come under the domain of malnutrition.^4^ Under nutrition puts children at higher risk of brutality and dying from common infections which also result in delayed recovery of school going children from diseases.^5^

School health facilities offer an ideal stage to identify the health problems early and treat them.^6^ Record of advanced countries has institutionalized school health programs as a vital portion of their education systems to recover the health circumstances and process of learning amongst school children.

This study was conducted to find out the prevalence of various nutritional issues in school children in rural areas of Islamabad. Lack of food supply has been one of the hurdles in child education which leads to lower admission in school in food deficient areas.^7^ Teenage high school children require nutrition rich diet so that it can uplift their physical, cognitive growth in the period of adolescence.^8^

School age is a dynamic period of physical growth as well as of mental development of the child. Nutritional status of school aged children effects their health and is considered a significant and positive health indicator. Good nutrition provides stronger immune system, better health and productivity.^9^

Pakistan is a country in transition and now faces double burden of coexistent over nutrition and undernutrition. Obesity is becoming an increasingly prevalent problem in Pakistan, as it has in other developing countries; with undernutrition remaining a problem simultaneously which has been observed in comparable developing countries such as Egypt and India.^10^ Our objective of the study was to determine the frequency of nutritional status and socio-demographic factors influencing under nutrition among school children of rural Islamabad.

## METHODS

This is a cross-sectional study conducted at schools of Bara Kahu, Islamabad as a part of School Health Program. Study period was from January 2017 to September 2019. As a part of School Health Program of Islamabad Medical and Dental College, intermittent screening visits were made to local schools. All 1710 students screened during this time period were included in the study. Schools were selected through convenient sampling technique. The data was collected from all the students of participating schools available at the time of visit. A structured questionnaire was used for data collection. A digital weighing scale was used for weighing children. For height, measuring tape was used. There was checking of zero error and calibration done in the instruments used. Frequency and percentages of categorical variables were calculated and inferential statistics were computed to analyze the association of health status with categorical variables by using chi-square by keeping level of significance <0.05 through SPSS version 20 (https://www.ibm.com/support/pages/downloading-ibm-spss-statistics-20)

The study was approved by the Institutional Review Board (dated September 25, 2017) of Islamabad Medical and Dental College, Islamabad. The schools were informed prior to the visits and informed consent was obtained from the parents through a consent note written on the homework diaries of the children.

## RESULTS

The data was collected from 1710 participants from different schools of Bara Kahu, Islamabad. Out of these 49.6% were males and 50.4% were females. Regarding type of schools 51.1% of the children belonged to private schools while 49.6% were studying in government schools. The mean age of the sample was 9.38 ± 4.14 with the maximum number of children (49.1% ) in the age bracket of 5-9. (Table-I).

**Table-I:** Demographic Characteristics of Study Participants.

		Frequency	Percentage (%)
Gender	Male	849	49.6
	Female	861	50.4
Age	0-4	141	8.2
	5-9	840	49.1
	10-14	507	29.6
	15 and above	222	13
Students Belonging to schools	Government	837	48.9
	Private	873	51.1

The nutritional status of 1710 children is shown in Fig-I. Frequency of stunting was found to be 444 (26%), which was calculated by plotting the heights of participants on growth chart and defining stunting as height for age <5^th^ percentile. While relating the nutritional status with age, malnutrition was observed in all age groups. Prevalence of being underweight was higher than overweight / obesity particularly in younger and higher age groups as indicated by p-value of 0.000. Likewise, gender was also found to be a strong factor influencing the nutritional status as while comparing with females, male students had significantly higher frequency of being underweight and stunting as reflected by p-value of 0.004 and 0.000 respectively. Considering the type of schools prevalence of stunting was more in private schools (Table-II)

**Fig.1:**
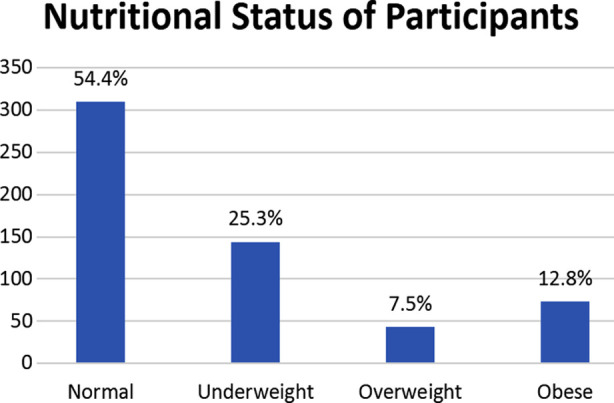
Nutritional Status of Study subjects based on CDC Recommendations.

**Table-II:** Association between Demographic Characteristics and Nutritional Status.

Variable		Nutritional Status					

		Normal	Underweight	Overweight/ Obese	p-value	Stunting	p-value
Age	0-4	48 (34%)	45 (31.9%)	48 (34%)	0.000*	33 (23.4%)	0.525
	5-9	417 (49.6%)	207 (24.6%)	216 (25.7%)		204 (24.3%)	
	10-14	315 (62.1%)	126 (24.9%)	66 (13%)		153 (30.2%)	
	≥15	150 (67.6%)	54 (25.3%)	18 (8.1%)		54 (24.3%)	
Gender	Male	438 (51.6%)	264 (31.1%)	147 (17.3%)	0.004*	279 (34.8%)	0.000^*^
	Female	492(57.1%)	168 (19.5%)	201 (23.3%)		165 (19.4%)	
Type of school	Private	468 (53.6%)	243 (27.8%)	162 (18.6%)	0.278	258 (29.6%)	0.029^*^
	Government	462 (55.2%)	189 (22.6%)	186 (22.2%)		186(22.0%)	

* p-value significant (<0.05).

Univariate analysis also showed a strong association between age and nutritional status as mean weight increased from 39.22 ±5.21 to 63.50± 4.66 and height from 35.67±5.76 to 113.73± 29.22 with advancing age. (Table-III) With reference to CDC parameters, significant difference was also observed between mean weights of males and females with a CI from -2.48 to 7.27. However, no association was observed between height and gender. (Table-III).

**Table-III:** Mean weight and height in different age groups and gender.

	Age Group	Mean Values	p-value	Gender	Mean Values	p-value
Mean Weight	0-4	39.22 ±5.21	0.000	Male	65.60 ± 33.25	0.007
	5-9	45.57±6.17				
	10-14	56.71± 5.81		Female	63.20 ± 25.66	
	15 and above	63.50± 4.66				
Mean Height	0-4	35.67±5.76	0.000	Male	51.32 ± 9.66	0.982
	5-9	47.04±10.30				
	10-14	79.52± 19.75		Female	50.04 ± 9.23	
	15 and above	113.73± 29.22				

## DISCUSSION

The present study was conducted to assess the nutritional status of school children. It revealed that prevalence of being underweight was higher than overweight or obesity particularly in higher age groups. Similarly stunting was also found in a considerable portion. The results are comparable with other national and international studies. Eze JN et al while assessing the nutritional status of Nigerian school children found that 9.3% were wasted while in our study the frequency of underweight children was 25.3%.^11^ In the current study with reference to age malnutrition was maximally observed in younger age group. The results are comparable with study by Yankanchi et al carried out in Vijapura Karnatka which showed that more than half of primary school children of urban areas were under weight and stunted and the under nutrition was mostly observed in students of grade II and grade III.^12^ Rosato et al observed that maximum malnutrition was present between age groups of 6 – 8 years with no significance relationship with gender.^13^ Karak P et al assessed the nutritional status of children between ages from 16 – 18 years of Bankura district in Bengal and it was observed that in in rural area prevalence of under nutrition was 65% with BMI 18.2 ± 2.87 and in urban areas it was 20.88 ± 3.04.^14^ The current study was although conducted in the only rural areas of Islamabad but results obtained by Huriochi et al in Cambodia also showed that children in urban areas have greater weight and height than those in rural areas with p value less than 0.01.^15^

In the present study gender was also found to be a strong factor influencing the nutritional status as while comparing with females, males students had significantly higher frequency of being underweight while stunting was more common in females. Similar results were observed in school children of Ahmadabad where measurements of both height and weight were found to be lower in males as compared to females in all age groups.^16^ However contrasting results were obtained in a national study conducted in Abbottabad where the percentage of female under nourished students (67.7) was more than the male students (55.4).^17^ Similarly while considering stunting, Malongane et al in the national nutritional program of South Africa found out that prevalence was more in boys (11.3%) than females (7.4%) however the difference was not significant with only few children showed > +1 SD.^18^

In contrast to current study where no association of under nutrition in children of private and public sector schools, the national level study conducted by Riaz et al showed a significant difference was observed between the school children of private schools (23.1kg±0.58) and public schools (21.3kg±0.61) with p value less than 0.05.^19^ However present study revealed that stunting was more prevalent in private schools again in contrast to the study by El-Sabely AA in an Egyptian study where the public sectors school children were more short statured than children belonging to private sector schools.^20^

### Limitations of the study

Assessment of school going children is very important in developing nations like Pakistan. A limitation of this study was that data was not taken from schools of urban Islamabad for comparison. In addition, use of different growth standards in various studies can lead to difference in frequency of undernutrition.

## CONCLUSION

Our study reports frequency of underweight children to be 25.3%, overweight 7.5% and obese 12.8%. The overall percentage of stunting was computed to be 26%. The results of the present study reveal that higher proportion of male students were underweight and stunted. It is important to strengthen school health services, emphasize on nutrition and health education and to design special easily understandable IEC material for children, their parents and teachers to improve child health in our country. There is urgent need to focus the attention of leaders and policy makers towards the double edged sword of malnutrition, particularly in rural areas of Islamabad.

### Authors’s Contribution:

**SF:** Conceived,designed the study and contributed in data collection.

**TJ & SZ:** Performed statistical analysis and manuscript writing.

**FR:** Did review, proof-reading and final approval of manuscript.

**SF and FR** are accountable for the accuracy and integrity of the research.
